# Is it possible to use specific IgE to single latex allergens to discriminate between spina patients with natural rubber latex allergy and sensitization?

**DOI:** 10.1186/1743-8454-7-S1-S17

**Published:** 2010-12-15

**Authors:** Reinhold Cremer, Hans-Peter Rihs, Angela Gaspar, Graca Pires, Monika Raulf-Heimsoth

**Affiliations:** 1 Children’s Hospital, Clinics of the City of Cologne, Amsterdamer Str. 59, 50735 Cologne, Germany; 2 Institute of Prevention and Occupational Medicine of the German Social Accident Insurance, Ruhr-University Bochum, Germany; 3 Immunoallergy Department, Dona Estefania Hospital, Lisbon, Portugal

## Background

Eleven characterized allergens of Hevea brasiliensis are available as recombinant proteins for in vitro IgE diagnosis. Hev b 2 and Hev b 13 are only suitable as native proteins. By testing patient sera against this panel of recombinant and native allergens of Hevea brasiliensis individual sensitization profiles and the relevance of single allergens can be assessed.

## Materials and methods

Sera of 30 spina bifida patients with specific IgE to NRL were tested for allergen-specific IgE antibodies (sIgE) to natural rubber latex (NRL) and 13 single Hev b allergens using the ImmunoCAP system. The results of the specific IgE values against single allergens were plotted (frequency of antibodies against the single allergen vs. the percentage of the total latex-specific IgE-response). Minor and major allergens for spina bifida patients sensitized or allergic against NRL could be identified.

## Results

Regarding all 30 spina bifida patients with sIgE Hev b 1, 2, 3, 5, 6.01 and 13 were identified as major Hev b allergens. In the patients without latex-related symptoms Hev b 2 and Hev b 6.01 were found only in small percentages of the latex-specific IgE response and low frequencies (minor allergens) whereas in patients with latex-related symptoms these allergens were found in high concentrations and frequencies. Hev b 5 represents the allergen with the highest percentage of the latex specific IgE response in all groups of patients, Hev b 1 is the allergen with the highest frequency of sensitization (about 80%) in all groups.

## Conclusions

Latex extracts for skin prick testing or in vitro allergosorbents should contain the major allergens Hev b 1, 2, 3, 5, 6.01 and 13. In spina bifida patients Hev b 6.01 and Hev b 2 could be useful to distinguish sensitized from allergic patients.

